# Anesthetic Management of a Cesarean Section for Preeclampsia in a Parturient With Stickler Syndrome: A Case Report

**DOI:** 10.7759/cureus.51190

**Published:** 2023-12-27

**Authors:** Kenichi Takechi, Mayuko Fujimoto, Ichiro Shimizu

**Affiliations:** 1 Department of Anesthesia, Matsuyama Red Cross Hospital, Matsuyama, JPN

**Keywords:** partial hellp syndrome, pierre robin sequence, preeclampsia, cesarean section (cs), stickler syndrome

## Abstract

Stickler syndrome is a connective tissue disease with the pathogenic involvement of procollagen genes. It is characterized by ocular and joint abnormalities, hearing loss, and midfacial hypoplasia. In Stickler syndrome, the Pierre Robin sequence is a possible complication.

A 30-year-old female was admitted at 33 weeks of gestation. She had a genetic diagnosis of Stickler syndrome type 1. The parturient was diagnosed with preeclampsia, and a decision was made to terminate the pregnancy via cesarean section. Combined spinal epidural anesthesia was planned. Pediatricians were included in the operating room in case of neonatal resuscitation. The mother's perioperative course was stable. The neonate needed directional positive airway pressure. He was strongly suspected of having Stickler syndrome.

For those with Stickler syndrome undergoing cesarean sections, the risk of a difficult airway must be considered for both the parturient and the neonate. Adequate staffing and collaboration among anesthesiologists, obstetricians, and pediatricians are crucial.

## Introduction

Stickler syndrome is a connective tissue disease with an autosomal dominant inheritance pattern in most cases due to the pathogenic involvement of procollagen genes, such as COL2A1 and COL11A1. It is considered a rare disease, with prevalence figures ranging from one to nine per 10,000 people and an approximate incidence rate of one in 7,500 to 9,000 neonates [1].

In Stickler syndrome, the Pierre Robin sequence (micrognathia, glossoptosis, and airway obstruction) is a possible complication; this includes a difficult airway [2]. Osteogenesis imperfecta associated with Stickler syndrome may also be a relative contraindication for neuraxial anesthesia [3]. Furthermore, because Stickler syndrome is mostly inherited as autosomal dominant, neonates have a 50% chance of having this condition [1]. However, there are few reports of the perinatal management of parturients with Stickler syndrome, especially in the anesthetic management of cesarean sections. We report the anesthetic management of a parturient with Stickler syndrome during an emergency cesarean section due to preeclampsia.

This manuscript adheres to the applicable EQUATOR (Enhancing the QUAlity and Transparency Of health Research) guidelines.

## Case presentation

A 30-year-old female (height: 146 cm, weight: 47 kg, gravida 3, para 0) with a chief complaint of headache and upper abdominal pain was admitted at 33 weeks of gestation. She had a history of amblyopia, hearing loss, and a genetic diagnosis of Stickler syndrome type 1 from her previous physician. She also had a history of postnatal respiratory distress that required tracheal intubation for two months. During pregnancy, both parents did not prefer the fetal genetic diagnosis. However, fetal echography showed findings suggestive of a cleft palate, which was suspected to have been inherited by the fetus.

On admission, the patient’s blood pressure was 194/120 mmHg. Laboratory tests showed a serum aspartate aminotransferase (AST) level of 103 U/L, a lactate dehydrogenase (LD) level of 484 U/L, and a platelet count of 16×10４/μL. In addition, a urinalysis indicated proteinuria grade 3 (Table 1).

**Table 1:** Laboratory findings at admission WBC: white blood cell; RBC: red blood cell; Hb: hemoglobin; Plt: platelet; AST: aspartate aminotransferase; ALT: alanine aminotransferase; LD: lactate dehydrogenase; PT-INR: prothrombin time-international normalized ratio; APTT: activated partial thromboplastin time

Test	Patient's result	Reference interval
WBC count	10750 /μL	33-86 /μL
RBC count	430×10^4^ /μL	386-492×10^4^ /μL
Hb	12.6 g/dL	11.6-14.8 g/dL
Plt count	16 ×10^4^ /μL	15-35×10^4^ /μL
AST	103 U/L	13-30 U/L
ALT	45 U/L	7-23 U/L
LD	484 U/L	124-222 U/L
PT-INR	0.86 INR	0.8-1.2 INR
APTT	27.3 sec	25-45 sec

The parturient was diagnosed with preeclampsia and partial hemolysis, elevated liver enzymes, and low platelet count (HELLP) syndrome [4] by the obstetrician. Despite the continuous administration of magnesium sulfate and nicardipine, the systolic blood pressure remained above 180 mmHg. Since the continuation of the pregnancy was considered dangerous for the parturient, a decision was made to terminate the pregnancy via cesarean section. The parturient’s platelet count was within the normal range on admission, and she had no history of bleeding tendency. A previous computed tomography scan showed only mild scoliosis and no abnormalities in the spinal canal. Combined spinal epidural anesthesia was planned for the parturient. In addition to the premature birth, the neonate was likely to have Stickler syndrome and the possibility of a compromised airway after delivery was considered. Two pediatricians were included in the operating room in case neonatal resuscitation was needed.

In the operating room, the parturient was placed in the right lateral decubitus position, and monitoring was initiated using an electrocardiogram, pulse oximetry, and noninvasive measuring of blood pressure. An epidural catheter was placed at the L1/2 interspace. A mixture of 2.4 mL of 0.5% hyperbaric bupivacaine and 0.15 mg of morphine was then administered into the subarachnoid space from the L4/5 interspace. After five minutes, cold hypoesthesia below the level of the Th4 dermatome area was confirmed, and the operation was commenced. After the administration of spinal anesthesia, her blood pressure stabilized at a systolic pressure of around 120 mmHg. Magnesium sulfate was administered continuously during the surgery. After the start of the surgery, continuous epidural anesthesia with 0.2% ropivacaine was started at 5 mL/hr.

A male neonate weighing 1.528 kg was delivered successfully 14 minutes after the start of the surgery. Immediately after birth, the neonate was examined by the pediatricians. The neonate was spontaneously breathing, did not require tracheal intubation, and was transferred to the neonatal intensive care unit (NICU). The first- and fifth-minute Apgar scores were six and eight, respectively. The total operation time was 87 minutes.

The mother's postoperative course was stable, and the epidural anesthesia catheter was removed on the first postoperative day. The parturient complained of a postdural puncture headache, but the symptoms resolved within a few days. No severe complications related to neuraxial anesthesia, such as epidural hematoma, were observed. The neonate was found to have apnea of prematurity in the neonatal intensive care unit (NICU) and needed directional positive airway pressure. He was also noted to have clubfoot, a cleft palate, and a small jaw, and was strongly suspected to have Stickler syndrome.

## Discussion

Stickler syndrome affects multiple organ systems. It is characterized by ocular and joint abnormalities, hearing loss, midfacial hypoplasia, and hypermobility. It is classified into three types, with Stickler syndrome type 1 being the most frequently occurring. The inheritance pattern of types 1 and 2 is autosomal dominant, with offspring having a 50% risk of inheriting the disease. Although penetrance is high, the clinical picture outside the eye is highly variable [1]. Since Stickler syndrome is a connective tissue disease, it is thought to increase the risk of preterm premature rupture of membranes and an incompetent cervix, leading to preterm delivery [5].

The most frequently reported factor for the anesthetic consideration of Stickler syndrome is the difficulty in securing the airway due to the occurrence of cleft palate and the Pierre Robin sequence. However, it is known that airway compromise due to the Pierre Robin sequence improves with growth [6], and the majority of patients with Stickler syndrome can be anesthetized safely with standard airway management, according to the report of a large case series on airway complications in anesthesia for Stickler syndrome [2]. On the other hand, in obstetric patients, general anesthesia is associated with an increased risk of airway obstruction (including upper airway edema) or failed tracheal intubation [7]. Thus, anesthesiologists should consider parturients with Stickler syndrome as having a high risk of a difficult airway.

In the present case, we used combined spinal and epidural anesthesia. Stickler syndrome is complicated by conditions such as scoliosis and osteogenesis imperfecta, which can be problematic for neuraxial anesthesia [3]. Although rare, Stickler syndrome has been reported to be associated with von Willebrand's disease [8], so a detailed physical examination and interview regarding bleeding tendency are important before administering neuraxial anesthesia. Our parturient was also complicated by partial HELLP syndrome. For cesarean sections with thrombocytopenia, general anesthesia is considered when the platelet count is below 7.5×104/μL [9]. Because the thrombocytopenia was mild in this case, we could utilize neuraxial anesthesia. However, if the thrombocytopenia had progressed to HELLP syndrome, the choice of anesthesia methods might have been more complicated.

The neonate was born prematurely and might have required neonatal resuscitation. Thus, pediatricians and a nurse were ready in the operating room. Given that Stickler syndrome type 1 is inherited dominantly, the neonate had a 50% chance of having Stickler syndrome and had a possibility of airway complications due to the Pierre Robin sequence. In a series of cases of Stickler syndrome, a history of cleft repair was present in almost 40% of type 1 Stickler syndrome patients, with the Pierre-Robin sequence being present in 13% of type 1 patients [2]. Securing the airway in the presence of the Pierre Robin sequence is most difficult during the neonatal period [6]. Therefore, for cesarean sections in patients with Stickler syndrome, the risk of a difficult airway should be considered not only for the parturient but also for the neonate. There is evidence that general anesthesia is associated with an increased need for neonatal resuscitation, especially in the fetus, which is already compromised in utero [10]. Thus, general anesthesia should be avoided as much as possible in premature cesarean sections for those with Stickler syndrome. In such cases, it is also important to ensure adequate preparation and resources for neonatal resuscitation, even in the case of neuraxial anesthesia.

## Conclusions

In conclusion, for those with Stickler syndrome undergoing cesarean section, the risk of a difficult airway must be considered for both the parturient and the neonate. Thus, avoiding general anesthesia and careful planning for safe neuraxial anesthesia is crucial. Such patients also require adequate staffing for neonatal resuscitation and collaboration among anesthesiologists, obstetricians, and pediatricians.
